# Alterations in the Rice Coleoptile Metabolome During Elongation Under Submergence Stress

**DOI:** 10.3390/ijms252413256

**Published:** 2024-12-10

**Authors:** Vladislav V. Yemelyanov, Roman K. Puzanskiy, Ekaterina M. Bogdanova, Sergey A. Vanisov, Anastasia A. Kirpichnikova, Maria O. Biktasheva, Zhanna M. Mukhina, Alexey L. Shavarda, Maria F. Shishova

**Affiliations:** 1 Department of Genetics and Biotechnology, Faculty of Biology, St. Petersburg State University, Universitetskaya em., 7/9, 199034 St. Petersburg, Russia; 2 Laboratory of Analytical Phytochemistry, Komarov Botanical Institute of the Russian Academy of Sciences, ul. Professora Popova, 2, 197376 St. Petersburg, Russia; puzansky@yandex.ru (R.K.P.);; 3 Department of Plant Physiology and Biochemistry, Faculty of Biology, St. Petersburg State University, Universitetskaya em., 7/9, 199034 St. Petersburg, Russia; 4 Laboratory of Biotechnology and Molecular Biology, Federal Rice Research Center, 3, Belozerny, 350921 Krasnodar, Russia

**Keywords:** submergence, coleoptile, elongation, metabolomics, adaptation, tolerance, *Oryza sativa*

## Abstract

Plants known as obligate aerobes developed different mechanisms to overcome the damage incurred under oxygen limitation. One of the survival strategies to have commonly appeared in hydrophytic plants is the escape strategy, which accelerates plant axial organs’ growth in order to escape hypoxic conditions as soon as possible. The present study aimed to distinguish the alterations in coleoptile elongation, viability and metabolic profiles in coleoptiles of slow- and fast-growing rice varieties. All the parameters were tested at 3, 5 and 7 days after sowing, to highlight changes during seedling development in normal and submerged conditions. The obtained results indicated that coleoptile elongation correlated with higher resistance to oxygen deprivation. GS-MS-based metabolic profiling indicated that coleoptiles of the fast-growing cultivar accumulated higher amounts of sugar phosphates, disaccharides, fatty acid derivatives and sterols, which are important for maintaining growth, membrane stability and viability. The slow-growing variety was characterized by a greater abundance of carboxylates, including lactate and phosphoric acid, indicating an energy crisis and cytosol acidification, leading to cell damage and low tolerance. Therefore, a metabolomics approach could be used for phenotyping (chemotyping) in the large-scale screening of newly developed varieties with higher tolerance to oxygen deprivation.

## 1. Introduction

Tolerance to oxygen deficiency in plant organisms that are obligate aerobes appeared during the evolution process in a number of hydrophytic plants. Two adaptation strategies can be distinguished: avoidance of an anaerobic environment and quiescence. They affect changes in plants at different levels of organization, from genomic to phenomic. The escape strategy (low oxygen escape syndrome, LOES) is characterized by accelerating the growth of axial plant organs, hyponastic leaf curvature, formation of aerenchyma, initiation of adventitious roots, transformation of leaf anatomy to improve gas diffusion, etc. [1,2,3,4,5,6]. As a result, shoots begin to actively transport air to the flooded parts of the plant. The avoidance strategy is most common among hydrophytes in conditions of constant flooding. The quiescence strategy (low oxygen quiescence syndrome, LOQS) differs by instigating the inhibition of growth and metabolism, which leads to a limitation of carbohydrate consumption and the production of toxic fermentation products [2,5]. This strategy enables plants to save resources and survive short periods of flash flooding. After the water level drops and normal aeration conditions return, plants restore their growth using the stored energy [5,7].

Rice (*Oryza sativa*) along with water chestnut (*Eleocharis dulcis*), water caltrop (*Trapa natans*), watercress (*Nasturtium officinale*) and wasabi (*Eutrema japonicum*) belongs to a short list of hydrophytic cultured plants. When a rice grain germinates in a hypoxic or anaerobic environment, the first thing to appear is a coleoptile—the juvenile organ of the plant, which is a modification of the leaf, closed in a tube. Upon reaching the surface of the water, the top of the coleoptile is destroyed, and the coleoptile itself turns into a “snorkel”, which allows the seedling to be provided with the necessary amount of oxygen. Consequently, the faster the coleoptiles grow, the shorter the period of damaging action of oxygen deprivation. Thus, it can be expected that the growth rate correlates with resistance to flooding.

Rice coleoptile length under anoxia was used to evaluate whether a correlation exists between this morphological (coleoptile length) parameter and other biochemical and molecular traits [8]. To determine the differences, 141 Italian and 23 Sri Lankan rice cultivars were screened. The conclusion was that only ethanol production correlated well with anoxic coleoptile elongation. The other tested biochemical or molecular parameters were not dependent on differences in the ability of the coleoptile to grow under a lack of oxygen. Based on these results, the expectation was that, at least in part, the coleoptile phenotype (coleoptile length) cross-links with the metabolome and reflects rice tolerance to oxygen deprivation.

Metabolic alterations are an important marker of plant adaptation to oxygen deprivation. The first metabolomic profiling of plants under hypoxia was performed on barrel medic seeds (*Medicago truncatula*) in 2006 [9]. A number of studies were carried out using various metabolomic platforms (GC-MS, LC-MS, CE-MS and NMR), which revealed significant differences between the metabolite profiles of flood-tolerant and flood-sensitive plants [10]. The applied sensitive technique revealed that the marker groups of metabolites included the central metabolism network. Substances that accumulated in organs and tissues of plants resistant to oxygen deficiency included metabolites of glycolysis and fermentation (pyruvate, lactate), organic acids of the Krebs cycle (succinate, fumarate), amino acids associated with glycolysis intermediates, as well as derivatives of aspartate and glutamate, metabolites of the gamma-aminobutyric acid shunt (GABA, 4-hydroxybutyrate) and other anaplerotic pathways of NAD(P)H reoxidation. In sensitive plants, either there was no up-regulation of the abundance of these compounds or it was rather short-term. Products of fermentation dominated in their profiles [10,11].

A number of studies were performed on rice as the model object. Based on the genotype of *O. sativa* var. *japonica* (variety M202), a near-isogenic line M202(*Sub1a*) was obtained, harboring the *Sub1a* gene and characterized by greater resistance than the parent variety [12]. The *Sub1a* gene regulates the LOQS strategy. Metabolic profiling of leaves of the lines revealed more differences than in the transcriptome. Under oxygen deficiency, both genotypes were characterized by sucrose depletion and an increase in glucose levels, more pronounced in the *Sub1a* line. No differences in trehalose metabolism were found. Krebs cycle metabolites were generally reduced in both forms (except citrate). Most amino acids, including GABA, accumulated to a greater extent in the *Sub1a* line, while Ala, Val, Thr, Ile, Glu and Asn were up-regulated in the wild type (M202). The results obtained allowed the conclusion that the presence of the *Sub1a* gene enhances the reactions of carbon and nitrogen metabolism involved in adaptation to hypoxia.

Metabolomic profiling of leaves of rice using the avoidance strategy was performed on cultivars T65 (conventional rice) and C9285 (deepwater rice) and the near-isogenic line NIL-12, derived from cultivar T65 and containing an introgressed fragment of chromosome 12 from C9285 harboring the *Snorkel1* and *2* genes regulating the LOES strategy [13]. Deepwater varieties (C9285 and NIL-12) showed increased levels of sucrose, trehalose, fructose, glucose, as well as other glycolytic metabolites (hexose phosphates, phosphoglycerate and PEP) when submerged, which was accompanied by a decrease in the starch content in the leaves. Lactate accumulated more in the wild type (T65). Krebs cycle metabolites (citrate and malate) accumulated predominantly in deepwater forms, as did succinate, the differences in the level of which were manifested during prolonged submergence. In leaves of the deepwater line NIL-12, compared to the wild-type parent rice variety (T65), the amino acid content was reduced both in the control and after 24 h of submergence, while the levels of alanine, GABA, glutamate and glutamine did not differ. Also, in the deepwater line NIL-12, the level of auxin conjugates with amino acids decreased during flooding.

The above data clearly demonstrate the high sensitivity of metabolic profiling to differences in the tolerance of rice seedlings and their organs to oxygen deficiency. This approach could be employed for further investigations in the search for a possible correlation between the growth of coleoptiles and metabolic adaptation to oxygen deficiency.

Recently, we screened 36 varieties and forms from the collection of the Federal Rice Research Center (Belozerny, Krasnodar, Russia) for coleoptile elongation, including 24 cultivars of Russian selection [14]. Russian rice is the northernmost growing on the globe. It is cultivated in Southern Russia (Kuban and Rostov regions, Dagestan) and Far East (Primorye). Russian rice varieties originated from *O. sativa* var. *japonica*. Not a single rice variety of Russian selection possesses the *Sub1a* allele, unless it was specially introgressed by breeders. The result of this screening was the selection of the fast-growing cv. Kuban 3 and the slow-growing rice cv. Amethyst as a contrasting pair, differing in coleoptile axial elongation.

The aim of this investigation was to compare coleoptile elongation, tolerance to submergence conditions and alteration in metabolic profiles of coleoptiles of slow- and fast-growing varieties in order to reveal marker groups of central metabolic substances characteristic of plants resistant to oxygen deficiency.

## 2. Results

### 2.1. Elongation of Rice Seedlings Under Submergence Conditions

**Figure 1:**
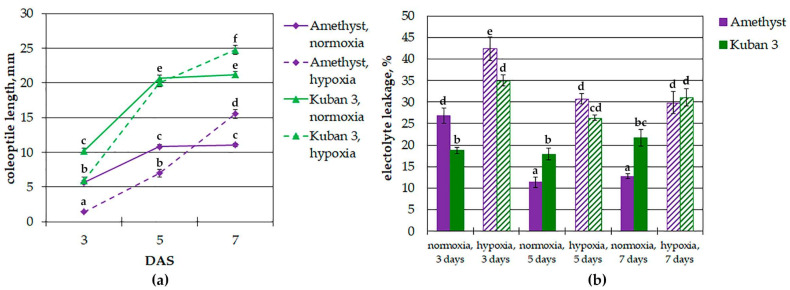
The length of (**a**) and electrolyte leakage from (**b**) the coleoptiles of the slow-growing Amethyst and the fast-growing Kuban 3 varieties of rice during normoxic and hypoxic germination. Values with the different letters are significantly different at *p* < 0.05, according to Tukey’s test. DAS—days after sowing.

The analysis of the alteration in the length of rice coleoptiles during normoxic and hypoxic germination is shown in Figure 1a. Two varieties were used in this study: the fast-growing Kuban 3 and the slow-growing Amethyst. It could be seen that under normoxic conditions, the coleoptiles of both varieties reached their maximum length on the fifth day of seedling development. However, the length of the coleoptiles of the Kuban 3 variety was two times greater than that of Amethyst. Then, the length of the coleoptiles did not change until the seventh day of seedling growth. With a lack of oxygen (under complete submergence), the elongation of coleoptiles was different. Note that on the third day of hypoxic germination, the coleoptile length was less than that of the control, regardless of the variety. The elongation of coleoptiles of the Kuban 3 variety intensified and reached the control (normoxic) level by the fifth day after sowing (DAS). In Amethyst, the length did not reach the control level by the fifth day. The continuation of coleoptile elongation was determined through further seedlings’ development of both varieties during submergence. The elongation of coleoptiles was completed on the eighth day of seedling development.

### 2.2. Viability of Rice Seedlings Under Submergence Conditions

To determine possible differences in the viability of rice seedlings of the slow- and fast-growing varieties, we measured the relative electrolyte leakage from coleoptiles germinating in normoxic and hypoxic environments (Figure 1b). An electrolyte leakage assay evaluates the integrity of cell membranes, where a higher leakage value corresponds to a higher degree of cell damage and less viability. Electrolyte leakage was different between coleoptiles of Amethyst and Kuban 3 on all days of normoxic germination; on 3 DAS, it was higher in Amethyst, and on 5–7 DAS, in Kuban 3. Oxygen deprivation resulted in stimulation of leakage from seedling coleoptiles (2-fold in Amethyst and 1.5-fold in Kuban 3). Under submergence, the viability of Amethyst coleoptiles was somewhat lower, and on 5–7 DAS, it was equal in both slow- and fast-growing varieties.

### 2.3. Metabolic Profiling of Rice Coleoptiles Under Submergence Conditions

**Figure 2:**
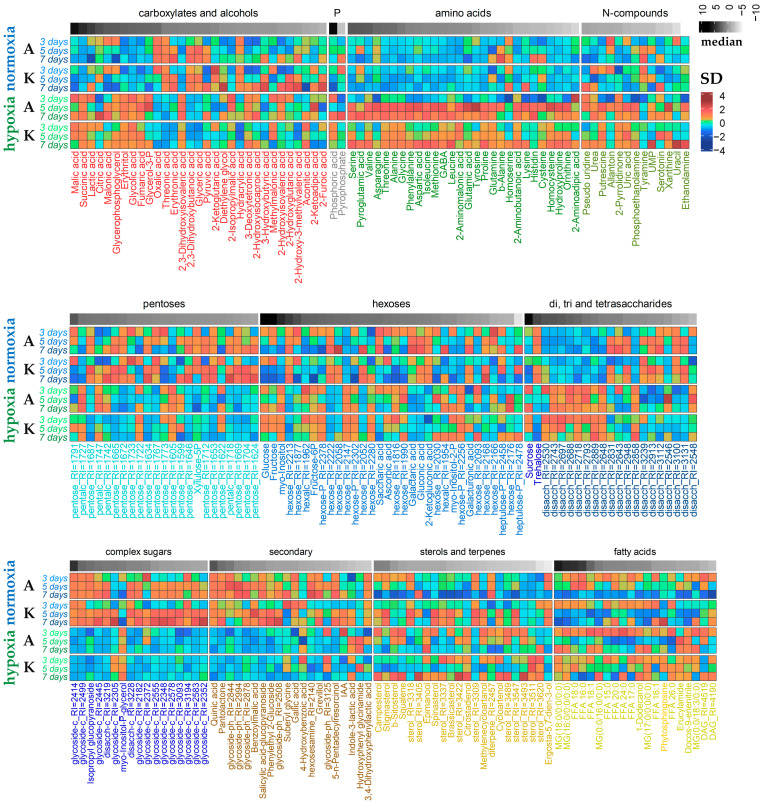
Heatmap of normalized and scaled mean metabolite contents in coleoptiles of slow-growing cv. Amethyst (A) and fast-growing cv. Kuban 3 (K) under normoxic and hypoxic germination. Metabolites were divided into groups by chemical properties. Gray annotation above is a heat map of the normalized median abundance. Metabolite key: RI—retention index, -P—phosphate, disacch—disaccharide, compsug—complex sugars or molecules with sugar parts (glycosides), FA—fatty acid, MG—monoacylglycerol.

The obtained metabolite profiles of rice coleoptiles included about 370 metabolites (Table S1), 230 of which were annotated (96 to individual compounds, 134 to classes, Figure 2). Sugars and their derivatives, including pentoses, hexoses, oligosaccharides and complex sugars (glycosides), were the most widely represented in the obtained profiles. Additionally, 28 amino acids, including 18 proteinogenic ones; about three dozen carboxylic acids, including intermediates of energy metabolism (3 from glycolysis and 6 from the Krebs cycle among them); and 20 free fatty acids and their derivatives, as well as nitrogenous bases (13), sterols (22), phenolic compounds (20), etc., were registered. The heat map illustrates the complex dynamics of metabolite accumulation in normoxic and hypoxic environments (Figure 2).

**Figure 3:**
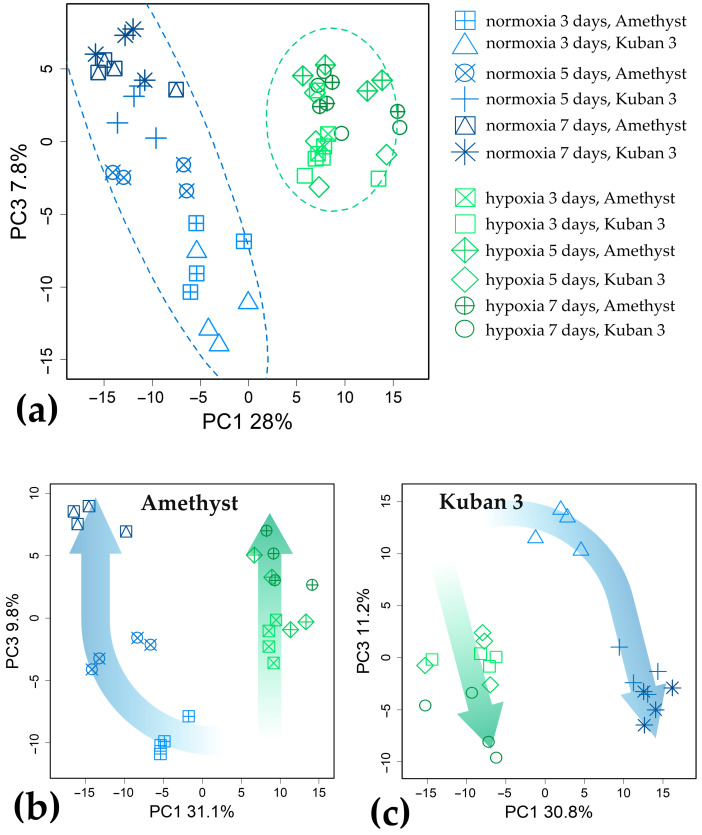
Unsupervised analysis of metabolite profiles from rice coleoptiles. Comparison of hypoxic action on both varieties (**a**), slow-growing cv. Amethyst (**b**) and fast-growing cv. Kuban 3 (**c**). PCA score plots. Ellipses are 95% confidence intervals.

For exploration and visualization of metabolomic data, the linear dimensionality reduction technique (principal component analysis, PCA) was used. In Figure 3a, the profiles of both cultivars are presented in the space of the first and third principal components (PCs). The metabolic profiles of the control and submerged plants form two separate groups that differ in PC1, explaining 28% of the variance. PC3 (8%) is associated with age differences in coleoptiles of control plants. At the same time, hypoxic plants form a dense group in PC3. Consideration of each variety separately showed different developmental trajectories under normoxia and hypoxia (Figure 3b,c). In both cases, plants of different ages varied under control conditions. For Amethyst, all three ages were clearly distinguished (Figure 3b), and in the case of Kuban 3, a jump occurred between 3 and 5 DAS (Figure 3c). Age differences were much weaker under hypoxia. This indicates arrested development under the action of hypoxia as a powerful metabolic driver. Since the age of plants seriously affected the metabolite profiles, we considered the differences between hypoxic and control plants at each point of germination separately.

#### 2.3.1. Metabolic Profiling of Rice Coleoptiles After 3 Days of Submergence

To reveal the details of the differences in the metabolic profiles of control and submerged plants, we compared the variants and classified each cultivar using the Orthogonal Partial Least Squares-Discriminant Analysis (OPLS-DA). The models included a predictive and an orthogonal component. The parameters of the models (Q^2^, R^2^, etc.) are given in Table S2. In the case of cv. Amethyst, the predictive component was associated with 40% of the variance, and in the case of cv. Kuban, 3–36%.

**Figure 4:**
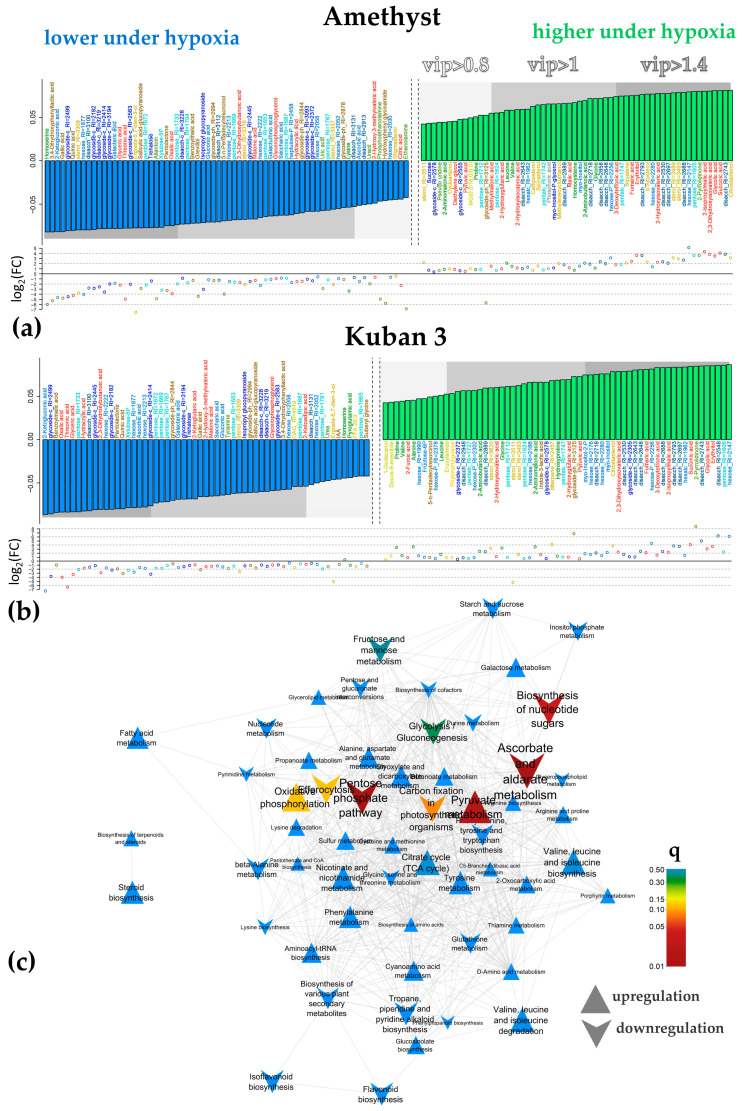
Differently accumulated metabolites under hypoxia after 3 days. Bar plots of factor loadings of the predictive components from OPLS-DA models for cv. Amethyst (**a**) and Kuban 3 (**b**). Scattered plot—log_2_(FC(hypoxia/normoxia)). (**c**)—Metabolite set enrichment analysis based on loadings from OPLS-DA classification for cv. Amethyst. Nodes are the paths extracted from KEGG. If the paths share metabolites, then they are connected by edges. Color—significance of influence on this pathway, size—strength of influence (|NES|), upward triangles—up-regulation under hypoxia, downward direction—down-regulation.

Factor loadings and VIP values were used to select differentially accumulating metabolites (DAMs). Oxygen deprivation promoted the accumulation of pyruvate and the Krebs cycle intermediates, except for citrate after 3 DAS (Figure 4a,b). Some other carboxylates, such as glycolate, also showed increased accumulation. At the same time, a number of carboxylic acids showed a decrease in their levels under hypoxia, including oxalate, glycerate, threonate and malonate. Separately, we noted trends toward a decrease in citrate levels in the case of Amethyst and 2-ketoglutarate for Kuban 3. Hypoxia also promoted the accumulation of amino acids (Gly, Leu, Pro, Val). Changes in the pattern of sterol accumulation were observed. For the most part, its accumulation increased under hypoxia. Disaccharides also showed some upward trends, while trehalose abundance on the contrary was down-regulated. The decrease in the level of sugar acids (ascorbic, galactaric, gluconic, 2-ketogluconic and saccharic) during hypoxia was noteworthy. Also, a decrease in a few secondary metabolites (mainly phenolics) was observed during hypoxia. A slight decrease in salicylate glycoside in both varieties and IAA in Kuban 3 is also worth mentioning.

To relate the data on alterations in metabolite accumulation, a metabolite set enrichment analysis (MSEA) was performed using factor loadings to rank metabolites and pathway sets obtained from the KEGG database. The data were rather similar, but they were more pronounced in the case of cv. Amethyst. Figure 4c shows the results of the analysis for this cultivar, clearly revealing that hypoxia suppressed the pentose phosphate pathway (PPP), accumulation of ascorbate and nucleotides. Additionally, submergence activated pyruvate metabolism.

To compare the effect of 3-day hypoxia on the two genotypes, Amethyst and Kuban 3, we compared the loadings of the predictive components from the corresponding OPLS-DA models (Figure S1a). We found that on the third day of germination, they demonstrated high similarity (rho = 69, *p* < 10^−15^, rho—Spearman’s correlation).

#### 2.3.2. Metabolic Profiling of Rice Coleoptiles After 5 Days of Submergence

**Figure 5:**
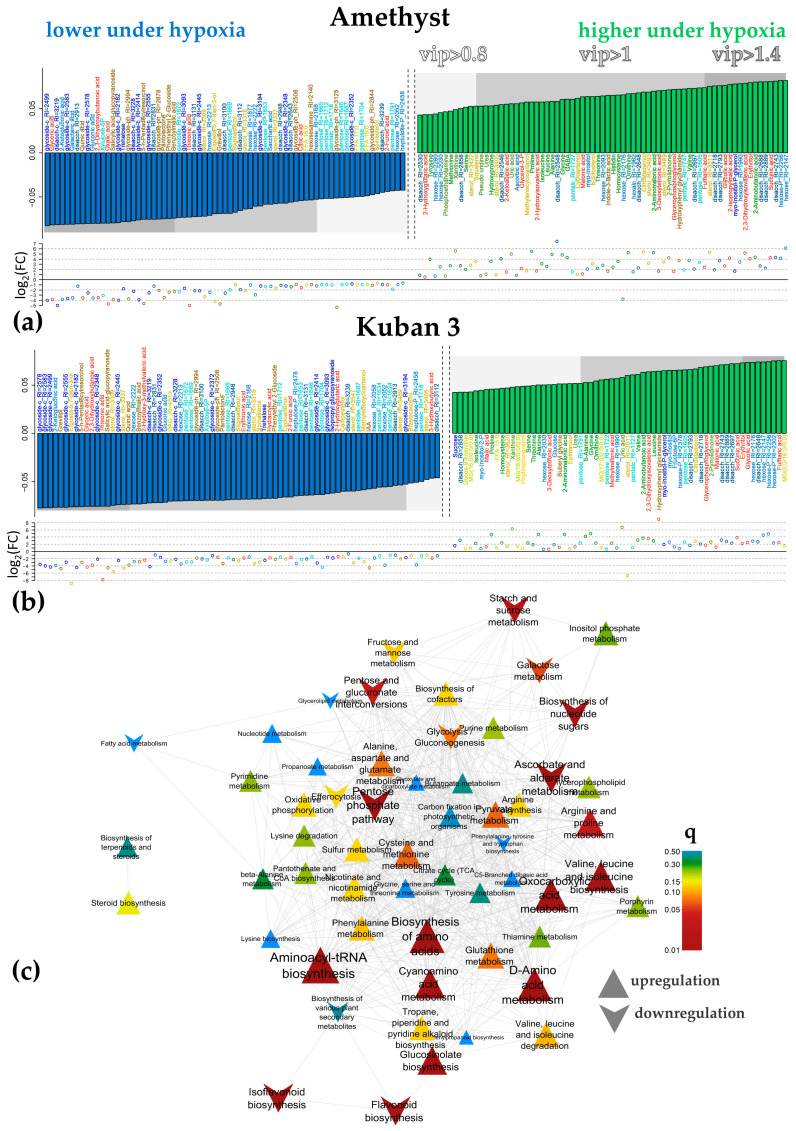
Differently accumulated metabolites under hypoxia after 5 days. Bar plots of factor loadings of the predictive components from OPLS-DA models for cv. Amethyst (**a**) and Kuban 3 (**b**). Scatter plot—log_2_(FC(hypoxia/normoxia)). (**c**)—Metabolite set enrichment analysis based on loadings from OPLS-DA classification for cv. Amethyst. Nodes are the paths extracted from KEGG. If the paths share metabolites, then they are connected by edges. Color—significance of influence on this pathway, size—strength of influence (|NES|), upward triangles—up-regulation under hypoxia, downward direction—down-regulation.

The predictive components accounted for 40% and 45% of variance for the Amethyst and Kuban 3 varieties in the 5-day hypoxic exposure OPLS-DA models (Table S2), respectively. Hypoxia promoted the accumulation of the Krebs cycle intermediates (succinate, fumarate and malate) after 5 DAS (Figure 5a,b). At the same time, some carboxylates showed a down-regulation (citrate, glycolate). A pronounced positive effect of submergence on amino acid (Ala, Gly, Ile, Leu, Orn, Thr, Val) abundance was observed in both cultivars, while Kuban 3 accumulated α-Ala, Pro and Ser. A decrease in stigmasterol and spinasterol levels could be noted in both cases, but changes in sterol metabolism were different in slow- and fast-growing cultivars. Eight sterols were up-regulated and six down-regulated in Amethyst, while there were three and twelve, respectively, in Kuban 3 (Figure 5a,b). It should be noted that monoacylglycerols (MGs) accumulated more in Kuban 3 too. As for 3 DAS, hypoxia led to a decrease in the levels of secondary compounds and sugar acids at 5 DAS. At the same time, the level of hexoses and their phosphates increased, particularly in Kuban 3 coleoptiles (Figure 5b).

Figure 5c shows the results of MSEA, demonstrating how different groups of metabolites were related to the effect of 5-day hypoxia. It can be seen that after 5 days of submergence, the effect of oxygen deprivation had expanded and affected a wider range of pathways. It should be noted that hypoxia repressed carbohydrate metabolism: sucrose and starch, pentose, hexose and sugar acid metabolism, PPP, glycolysis and gluconeogenesis, etc. The effects were more pronounced in Amethyst. At the same time, hypoxia increased the accumulation of intermediates of pyruvate metabolism, oxocarboxylates, and activated various amino acid metabolism pathways. Separately, it is necessary to mention the repression of flavonoid metabolism. Again, the decrease in salicylate glycoside in both varieties and IAA in Kuban 3 was observed.

A comparative analysis of the effect of submergence on the two varieties showed that at 5 DAS, they exhibited high similarity, slightly higher than at the previous stage (rho = 0.72, *p* < 10^−16^). The differences were associated mainly with the group of sterols and fatty acid derivatives (Figure S1). A comparison of the effects of hypoxia on 3 and 5 DAS showed their significant similarity (rho = 0.63 and 0.55 for Amethyst and Kuban 3 respectively, Figure S2). The differences in the case of Amethyst were related to amino acids, whose accumulation was more greatly stimulated by hypoxia at 5 DAS. Differences also concerned the effects of submergence on sterols, glycosides and a small number of carboxylates, such as malonate.

#### 2.3.3. Metabolic Profiling of Rice Coleoptiles After 7 Days of Submergence

**Figure 6:**
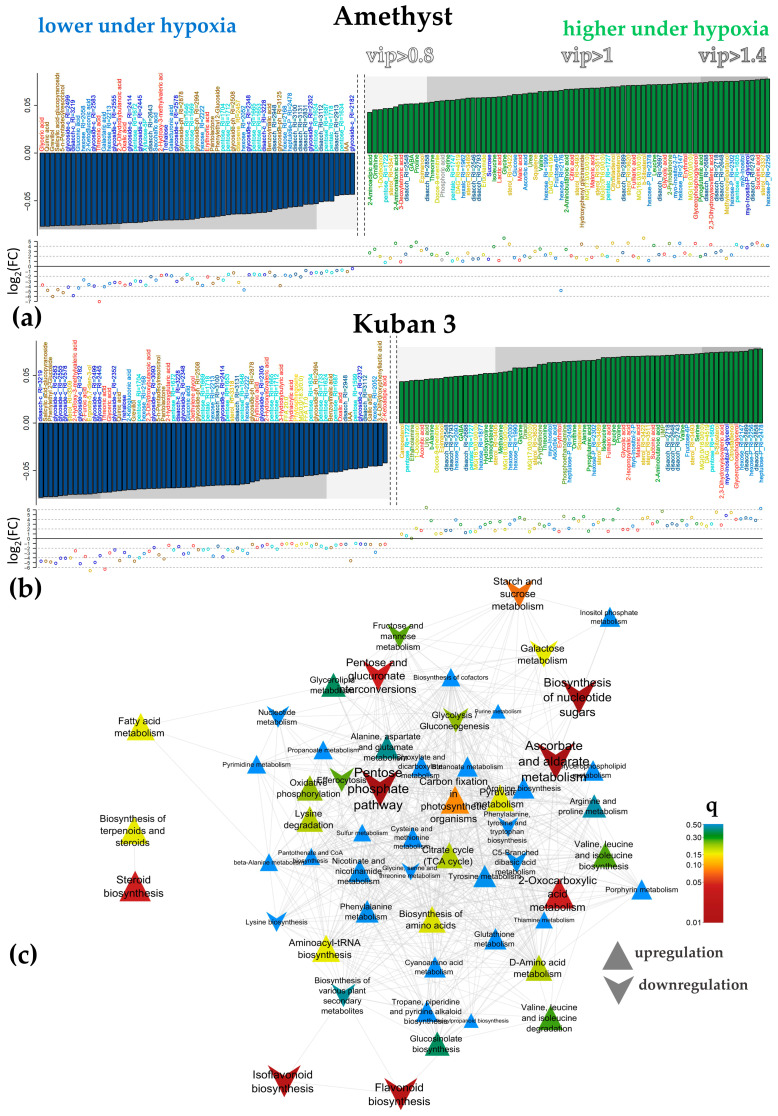
Differently accumulated metabolites under hypoxia after 7 days. Bar plots of factor loadings of the predictive components from OPLS-DA models for cv. Amethyst (**a**) and Kuban 3 (**b**). Scatter plot—log_2_(FC(hypoxia/normoxia)). (**c**)—Metabolite set enrichment analysis based on loadings from OPLS-DA classification for cv. Amethyst. Nodes are the paths extracted from KEGG. If the paths share metabolites, then they are connected by edges. Color—significance of influence on this pathway, size—strength of influence (|NES|), upward triangles—up-regulation under hypoxia, downward direction—down-regulation.

In the models for the 7-day exposure, the predictive components accounted for 46% and 43% of the variance for the Amethyst and Kuban 3 varieties, respectively (Table S2). Again, we can see that carboxylates showed an increase in abundance, but there were nuances. Hypoxia promoted the accumulation of the Krebs cycle intermediates (malate, succinate and fumarate) as well as glycolate and malonate in both genotypes after 7 DAS (Figure 6a,b). At the same time, some carboxylates showed a decrease in accumulation, among which we noted a decrease in the levels of glycerate, threonate and oxalate. Differences were found in the levels of lactate and malate, which were increased in Amethyst coleoptiles (Figure 6a). Kuban 3 was characterized by the accumulation of aconitate, while 2-hydroxyglutarate and other 2-hydroxycarboxilic acids were down-regulated (Figure 6b). A positive effect of hypoxia on the abundance of amino acids was observed. In particular, Gly, GABA, Ile, Leu, Orn, Pro, Ser, Val and pyroglutamate (oxoproline) showed trends towards accumulation in both varieties, whereas α- and β-Ala, along with Met, were up-regulated in Kuban 3. Oxygen deficiency promoted the increase in levels of lipophilic compounds (lysolipids, sterols and squalene). Again, hypoxia led to a decrease in the abundance of phenolic compounds, glycosides and sugar acids, particularly in Amethyst coleoptiles (Figure 6a). The ascorbate level was up-regulated as well as the level of hexoses and their phosphates, while pentoses and trehalose amounts were depleted. A decrease in IAA abundance was also noted (Figure 6a,b).

MSEA demonstrated trends similar to those observed early with the 5-day submergence, but many of them were less significant (Figure 6c). Of the new ones, we noted the stimulation of the metabolism of lipophilic compounds: sterols, terpenoids and fatty acids.

A comparative analysis of the effects of oxygen deprivation on two varieties showed that at 7 DAS of submergence, they exhibited high similarity (Figure S1), slightly higher than at 5 DAS (rho = 0.78, *p* < 10^−16^). Thus, the similarity of the metabolic response gradually increased. The differences were primarily associated with the group of sterols and fatty acid derivatives, as in the previous case. A comparison of the effects at 5 and 7 days of hypoxia showed their high similarity (rho = 0.77 and 0.73 for Amethyst and Kuban 3, respectively, Figure S2). The differences were related in the case of Amethyst to lipid metabolism and to a lesser extent to carboxylates and amino acids.

#### 2.3.4. Differences Between Varieties Under Hypoxia

**Figure 7:**
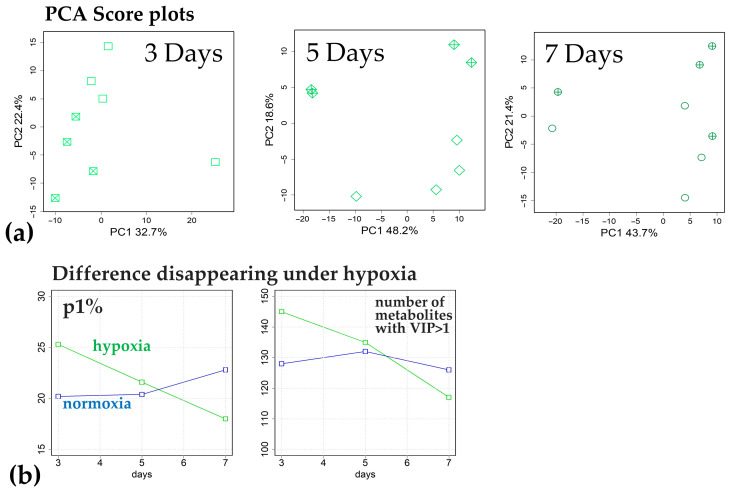
Metabolic differences between cultivars Amethyst and Kuban 3 under hypoxia. (**a**)—PCA score plots at 3, 5 and 7 DAS; (**b**)—OPLS-DA model parameters, illustrating leveling of intracultivar differences in hypoxia, but not in normoxia. p1%—percent of variance, related to predictive component.

Since the metabolic profiling of coleoptiles of the two rice varieties showed differences under hypoxia, we compared them under these conditions. PCA revealed that coleoptiles of the two varieties clearly differed at 3 and 5 days of hypoxia in the space of the first two PCs (Figure 7a). The dissimilarities were less pronounced in coleoptiles of 7-day-old plants. OPLS-DA modeling showed that the proportion of variance associated with varietal differences (Table S2) decreased with time, as did the predictive power of the predictive component and the number of differentially accumulated metabolites (Figure 7b). However, this did not happen with the models for plants under normoxia. Thus, it could be argued that germination and growth under hypoxic conditions leveled out the differences between coleoptiles of the two varieties. In addition, it should be noted that intervarietal differences were relatively weak compared to the effect of hypoxic stress.

**Figure 8:**
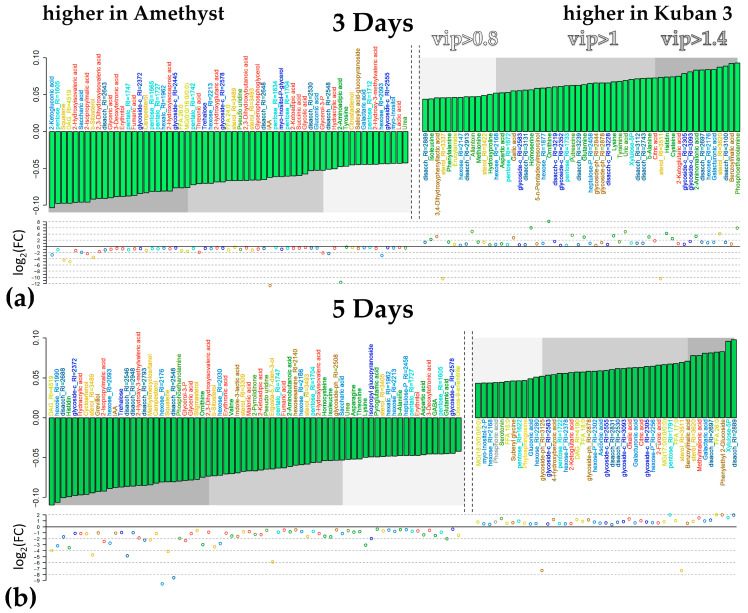
Metabolites differently accumulated in cv. Amethyst and cv. Kuban 3 under 3 (**a**) and 5 (**b**) days of hypoxia in two cultivars. Bar plots of factor loadings of the predictive components from OPLS-DA models. Scatter plot—log_2_(FC(hypoxia/normoxia)).

Comparison of the metabolomes of the two varieties after 3 and 5 DAS revealed that the slow-growing variety Amethyst when submerged differed from the fast-growing Kuban 3 in having a greater accumulation of carboxylates, including intermediates of the dicarboxylic part of the Krebs cycle (succinate and fumarate), glycolate, glycerate, lactate and 2-hydroxycarboxylates (Figure 8a). At the same time, the initial intermediates citrate and 2-ketoglutarate showed a higher level in Kuban 3. The difference in the level of amino acid accumulation was more complex. Amino acids showed a tendency to accumulate more in the coleoptiles of Kuban 3 at 3 DAS. Among, them sulfur-containing amino acids (Cys, Met), Asp, Gln, His, Ile, Lys and β-Ala could be distinguished. On the fifth day of submergence, the differences in the amino acid content changed almost to the opposite (β-Ala, Asp, Asn, GABA, Glu, His, Ile, Lys, Thr and Val dominated in Amethyst coleoptiles, Figure 8b). The picture also changed in the case of lipophilic compounds. If Amethyst showed a greater relative accumulation of fatty acids and acylglycerols at 3 DAS, then on 5 DAS, this was characteristic of Kuban 3. The pattern of sterol abundance also differed significantly. It was noted that Amethyst accumulated major sterols (10 compounds) more on the fifth day of submergence. At 7 DAS, the differences between the varieties were reduced to a slightly higher accumulation of sterols (16 compounds) in the coleoptiles of Kuban 3 (Figure S3a). Differences in the accumulation pattern of other compounds were also observed. The levels of IAA and trehalose in Amethyst were higher than in Kuban 3 at all time points of submergence. Kuban 3 coleoptiles were characterized by greater levels of sugar phosphates, disaccharides and glycosides. Varietal distinctions were most significant on the third and fifth days of flooding, but the varieties retained some common features (Figure S3b, rho = 0.33, *p* = 10^−10^). Also, a comparison of intervarietal differences under normoxia and hypoxia revealed only a moderate relationship between them, if any (Figure S3c). The latter suggests that the effect of hypoxia is the most important factor determining the implementation of the genotype in the biochemical phenotype (chemotype).

#### 2.3.5. The Influence of Hypoxia on the Correlation Links Between Metabolite Pools

**Figure 9:**
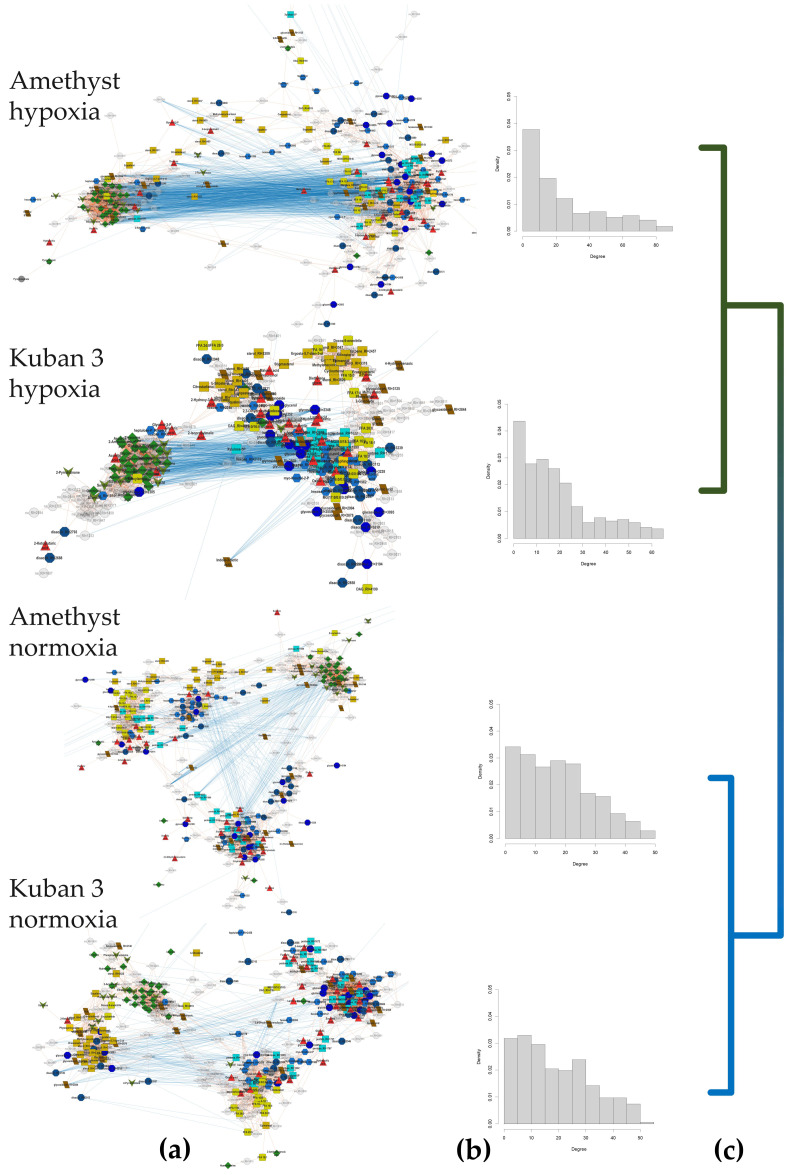
Mapping of metabolites by correlations of contents (Pearson’s correlation, q < 0.05) (**a**); histograms of node degree distributions (**b**); dendrogram of hierarchical clustering by the similarity of edge sets (**c**).

To identify the effects of submergence on the functional connectivity of metabolite pools, we mapped metabolites by their normalized abundance correlations at each experimental time point (Figure 9a). Plots with nodes representing metabolites and edges representing significant correlations (q < 0.05) were constructed such that positive connections attracted metabolites proportional to the correlation value. The networks varied in both appearance and characteristics (Table S3). However, common features were found between them. The networks demonstrated heterogeneity. Clusters of metabolically related compounds such as amino acids, sterols, fatty acids and acylglyceroles were observed. Carboxylates were scattered throughout the network. In addition, all networks had more positive connections than negative ones, with the ratio of positive to negative decreasing under hypoxia. Amethyst also showed a significant increase in the number of correlations under oxygen shortage. It can be noted that under submergence, the diameter of networks slightly increased. Also, there was some increase in parameters such as density, heterogeneity and centralization under oxygen deprivation. The effect of hypoxia was not only the increase in the number of connections but also the change in their distribution, as can be seen from the histograms in Figure 9b. Under submergence, the distribution became more similar to a power law. The number of metabolites with a small number of connections increased, and on the other hand, a “tail” made up of a small number of metabolites with an increased number of connections appeared. In order to determine the similarity of the structure of functional connections, we clustered the networks by similarity. First, we compared the networks by the similarity of node degrees (Bray–Curtis dissimilarity). Second, we compared the networks by the composition of edges (pairs of metabolites, Jaccard index). The networks were clustered (Ward method), and the resulting dendrograms for the two comparison methods were similar (Figure 9c). As it turned out, the networks for hypoxic coleoptiles of the two varieties were separated from those under normoxia. Thus, hypoxia leads to significant reorganizations of the functional connections of metabolite pools.

## 3. Discussion

Alteration of growth is one of the important components of the adaptive response of plant organisms to submergence and, consequently, to oxygen deficiency. The strategy of escape from oxygen deprivation (low oxygen escape syndrome, LOES) results in stimulation of shoot growth, whereas the quiescence strategy (low oxygen quiescence syndrome, LOQS) is characterized by growth inhibition. Acceleration of the growth rate during submergence has been demonstrated in a number of plants, and at different stages of development: in *Callitriche platycarpa* and *Nymphoides peltata* [15], deepwater rice (*O. sativa*) [16], *Potamageton pectinatus* [17], *Ranunculus sceleratus* [18], *Rorippa amphibia* and *Rumex palustris* [19], tropical fern (*Regnellidium diphyllum*) [20], etc. Growth arrest as a manifestation of the quiescence strategy is typical for *Arabidopsis thaliana*, *Oenathe aquatic*, Indian *Sub1a* rice varieties, *Rorippa sylvestris*, etc. [16,19].

Therefore, in hydrophytes, and particularly in rice, shoot growth, could reflect an adaptation strategy. Amethyst and Kuban 3 were chosen as a contrasting pair of rice varieties according to their differences in coleoptile elongation [14]. Comparative analysis of submergence effects on coleoptile length carried out in the present study confirmed the fast-growing properties of the Kuban 3 cultivar. The growth rate was about 5 mm a day in both normoxic and hypoxic environments at the active elongation stage (up to 5 DAS in control and 7 DAS when submerged, Figure 1a). Slow-growing cv. Amethyst manifested a coleoptile elongation rate of 2.5 mm a day in the case of normoxia and 3.5 mm under oxygen shortage.

In spite of the Amethyst and Kuban 3 cultivars significantly differing in coleoptile elongation, their viability was comparable (Figure 1b). The relative electrolyte leakage test demonstrated equal results at 5 and 7 DAS under submergence; only at 3 DAS did Kuban 3 coleoptiles show slightly but statistically significantly higher viability than Amethyst ones. Moreover, the hypoxia-induced increase in electrolyte leakage compared to controls was higher in cv. Amethyst (2-fold vs. 1.5). So, we surmise that Kuban 3 was slightly more tolerant to oxygen shortage than Amethyst. Surprisingly, the coleoptile viability of the studied cultivars differed from those of the control variants at all stages of the experiment, and in Kuban 3, it did not change at any time points (3–7 DAS), while in Amethyst, it increased. Thus, the Kuban 3 cultivar has a greater coleoptile elongation rate and a little more tolerance to submergence compared to the Amethyst one.

It is well-known that any changes in the intensity and direction of biochemical pathways lead to alterations in the metabolic profiles—dynamically changing parameters that summarize the entire spectrum of metabolites and characterize the state of a living system depending on the phase of development and the action of stress factors. In our work, we conducted a comparative analysis of the metabolic profiles of rice coleoptiles of two varieties that differ in elongation rate under normoxia and hypoxia (Figure 2, Figure 3, Figure 4, Figure 5, Figure 6, Figure 7 and Figure 8).

We used untargeted GC-MS-based metabolomics that allow for detecting alterations to the central metabolism, including the abundance of organic, amino and fatty acids, carbohydrates, sterols, a limited number of secondary compounds (terpenes, phenolics) and glycosides. For precise profiling of secondary metabolites, glycosides and sugars, an LC-MS metabolomic platform or targeted studies are more suitable. No platforms used for metabolic profiling are applicable to the detection of ethanol fermentation metabolites (acetic aldehyde, ethanol).

Our data indicated that Amethyst had stronger age-dependent differences in metabolomes under aerobic germination: metabolic profiles of coleoptiles were clearly separated from each other on 3, 5 and 7 DAS (Figure 3b). Although normoxic elongation of coleoptiles of both varieties was completed at between 5 and 7 DAS (Figure 1a), it seems that Kuban 3 coleoptiles developed faster and almost completely stopped in the time up to 5 DAS, since metabolic profiles at 5 and 7 DAS grouped together and clearly differed from 3 DAS (Figure 3c). Oxygen deprivation smoothed out age-related differences in both cultivars (Figure 3).

Submergence altered the metabolomes of both varieties in a rather similar way. It led to the accumulation of a number of organic acids associated with glycolysis and fermentation (pyruvate, lactate) and the Krebs cycle (succinate, fumarate); phosphoric acid and amino acids, particularly related to glycolysis and the GABA shunt (Ala, GABA, Gly, Ile, Leu, Pro, Val); nitrogenous compounds (bases, nucleosides); hexoses and hexose phosphates, sucrose, a number of oligosaccharides and ascorbate; squalene; and fatty acid derivatives. The levels of pentoses, trehalose, glycosides and phenolic compounds, on the contrary, were down-regulated (Figure 2, Figure 4, Figure 5 and Figure 6). Thus, the metabolic response of rice coleoptiles of both varieties was similar to that reported earlier for rice coleoptiles [11], shoots [12,13] and organs of other hypoxia-tolerant plants [10,21,22] and corresponded to the stimulation of anaerobic respiration, nitrogen metabolism and alternative pathways of NAD(P)H reoxidation reviewed in [10]. Nevertheless, our comparison of metabolic responses in coleoptiles of two rice varieties that differed in elongation rate revealed distinctions under oxygen limitation (Figure 7). The slow-growing variety Amethyst was characterized by a greater abundance of carboxylates, including glycolate, glycerate, lactate, intermediates of the dicarboxylic part of the Krebs cycle (succinate and fumarate) and 2-hydroxycarboxylates, as well as phosphoric acid (Figure 2 and Figure 8). Accumulation of lactate, inorganic phosphate and 2-hydroxycarboxylates may participate in cytosol acidification in plants under a lack of oxygen [2,6,10]. Phosphate and pyrophosphate accumulation was shown in Amethyst at 7 DAS under submergence, indicating a developing energy crisis (Figure 2 and Figure S3a). Also, the abundance of sugar phosphates, oligosaccharides and glycosides in the slow-growing cultivar was lesser, whereas the trehalose level was higher than in the fast-growing cultivar (Figure 2, Figure 8 and Figure S3a). Moreover, MSEA showed increasing suppression of carbohydrate and respiratory metabolism (glycolysis, PPP, sugar interconversion, starch digestion, etc.; Figure 4c, Figure 5c and Figure 6c) over time (from 3 to 7 DAS). Altogether, these processes could result in more damage and a lower growth intensity of Amethyst (Figure 1). On the contrary, depressed energy metabolism may lead to slow growth, as in the case of the LOQS strategy. But it is necessary to note again that Russian-bred rice varieties, including Amethyst, do not possess the *Sub1a* gene. The predominant accumulation of lactate also testified to the lower tolerance of slow-growing rice.

The fast-growing Kuban 3 variety was characterized by the accumulation of tricarboxylic acids of the Krebs cycle (citrate, aconitate, 2-ketoglutarate), sugar phosphates, disaccharides and glycosides during submergence (Figure 2 and Figure 8), similar to deepwater rice [13], indicating less suppression of carbohydrate and respiratory metabolism. There was no significant accumulation of lactate and 2-hydroxycarboxylates, corresponding to less intoxication by fermentation products. Also, the levels of fatty acid derivatives and sterols were elevated in Kuban 3 coleoptiles, particularly at 7 DAS under submergence (Figure 2 and Figure S3a). Changes in sterol composition crucially affected membrane fluidity and functional activity [6], reflecting alterations involved in growth processes.

It is important to note that trehalose abundance was down-regulated by oxygen shortage in both cultivars (Figure 4, Figure 5 and Figure 6), but intervarietal comparison detected a predominance of this disaccharide in coleoptiles of slow-growing Amethyst compared to Kuban 3 (Figure 8). Trehalose itself is involved in the stress response as an osmolyte, antioxidant and growth suppressor [23]. The trehalose precursor, trehalose-6-phosphate, inhibits the activity of hexokinase [23] and Sucrose-non-fermenting1-related kinase 1 (SnRK1), which act as sensors of the energy level and are activated by sugar depletion and, under conditions of energy deficit, including hypoxia, trigger starch mobilization [24]. Accumulation of trehalose-6-phosphate would suppress carbohydrate and energy metabolism, as we could see in Amethyst under submergence.

One more interesting finding concerned the level of phytohormone auxin (indole-3-acetic acid, IAA), which we found to be up-regulated in coleoptiles of slow-growing cv. Amethyst but not in fast-growing cv. Kuban 3 throughout all time points of submergence (3–7 DAS, Figure 8 and Figure S3a). Auxin was discovered to be a plant growth substance stimulating coleoptile elongation [25]. According to “acid growth theory”, in normoxic conditions, IAA activates plasmalemma H^+^-ATPases, which acidify cell walls. This, in turn, leads to the activation of a number of apoplast proteins that loosen the interaction between cell wall polysaccharides and ensure elongation growth [25,26,27]. The role of auxin in the regulation of coleoptile elongation under submergence is ambiguous. There are contrasting data on the accumulation and growth-promoting activity of auxin in rice oxygen-depleted coleoptiles [28]. The up-regulation of the IAA level in coleoptiles of both flood-tolerant cultivar C11 and -intolerant KY60 was revealed previously after flooding stress, but it was not significant [29]. Nevertheless, IAA accumulation in the tissues of more mature rice seedlings and other hydrophytes may suppress growth, as is known to occur for roots [30,31]. Moreover, high concentrations of auxin will stimulate ethylene production, which is known to be important for different adaptation mechanisms involved in plant tolerance to oxygen deprivation [32]. The identified phenomenon is also important because, besides growth regulation, IAA is involved in metabolic regulation during oxygen deficiency [31].

An important aspect of systems biology research is the analysis of relationships between components. Metabolite correlation can be used to assess the relationships of metabolites [33,34,35]. Correlation patterns have been shown to be specific to organs and tissues [36,37], to genotypes (including point mutations) [37] and to environmental conditions [38,39,40]. To reveal the effects of hypoxia and cultivar traits on the interconnected structure of metabolite pools, we mapped metabolites by their correlations. The networks differed in both visual appearance (Figure 9a) and characteristics (Table S3). This indicated significant functional differences among the variants. The networks were characterized by heterogeneity, with metabolically related compounds often appearing close to each other. In the case of hypoxia, the number of clusters was reduced. However, the cluster containing nitrogen-containing compounds (green nodes) remained and even became more clearly separated. At the same time, some carboxylates (red) were also close to it. This may be the result of the specific role of alterations in coordinated changes in amino acid metabolism during adaptation to hypoxia.

A prominent effect of hypoxia was the decrease in the ratio of positive and negative correlations by a factor of 2.9 and 2.4 for Amethyst and Kuban 3, respectively (Table S3). The increase in the proportion of negative correlations may be the result of reduced metabolic flux and the lack of energy and carbon [41,42]. In this case, it is not surprising that slow-growing Amethyst showed a greater decrease in the ratio of positive to negative correlations. It is also interesting that Amethyst showed an increase in the total number of significant correlations. Thus, a greater level of correlation can be considered a feature of more severe stress. The same hypoxia led to a change in the distribution of node degrees (Figure 9b). The number of low-degree nodes (metabolites with a small number of correlations) increased in the distribution and a long tail appeared (metabolites with a large number of correlations). This may be the result of the origin of new metabolic hubs providing an adaptation to stress. Clustering of variants based on the comparison of pairwise correlations (Figure 9c) showed that submergence was a more important determinant of metabolite relationships than cultivar specificities. This was consistent with the data on metabolite accumulation (Figure 3).

## 4. Materials and Methods

### 4.1. Plant Material, Growing Conditions and Imposition of Submergence Conditions

The seeds of two rice (*Oryza sativa* L. var. *japonica* auct.) varieties of Russian selection differing in coleoptile elongation from the collection of the Federal Rice Research Center (Belozerny, Krasnodar, Russia) were used in this study. The slow-growing rice cv. Amethyst and the fast-growing cv. Kuban 3 were chosen as a contrasting pair after screening 36 varieties and forms [14]. The caryopses were surface-sterilized with a 50% sodium hypochlorite solution for 15 min, washed 10 times with sterile water and soaked in hot water (55 °C) for 1 h. Then, 50 seeds of the control samples were placed in a tray on glass rows covered with gauze [14]. We poured 4% Knop nutrient solution [31] into the tray to the level of the glass rows, covered them with glass and germinated the seeds under conditions of normal air access. To create hypoxia, 50 seeds of the experimental variants were placed in 750 mL containers, submerged with the same solution to the very top (water column height 12 cm) and hermetically sealed with a lid. In both variants, the plants were grown for 7 days at 29 °C in the dark. Samples for analysis of coleoptile length, viability and metabolic profiling were taken at 3, 5 and 7 DAS. The oxygen content in the hypoxic solution did not exceed 0.6 ± 0.05 mg/L at all points of the experiment tested, as measured using the Expert-009 dissolved oxygen analyzer (Econix-Expert, Moscow, Russia). The dishes, gauze and solutions for experiments with plants were pre-sterilized.

### 4.2. Coleoptile Length Measurement

To measure the coleoptile length, the seedlings were placed in Petri dishes and scanned using an HP ScanJet G2710 (Hewlett-Packard, Palo Alto, CA, USA), and the images were digitized using ImageJ (version 1.8.0_172) (https://imagej.net/). All germinated seedlings out of the 50 sown were used for the analysis [14].

### 4.3. Electrolyte Leakage Test

Electrolyte leakage was measured from 10 coleoptiles collected from normoxic or hypoxic variants, as described earlier [31], using conductivity meter HI2300 (Hanna Instruments, Smithfield, RI, USA).

### 4.4. Sample Preparation for Metabolic Profiling

Plant material (0.2 g of coleoptiles) was collected in microtubes, frozen with liquid nitrogen and ground, as described previously [43]. Metabolites were extracted with 1 mL of methanol for 1 h in a thermoshaker TS-100C (BioSan, Riga, Latvia) at 800 rpm and 4 °C. After extraction, the samples were centrifuged for 10 min at 12,000× *g* and 4 °C, and then the residue was washed twice with 500 μL of methanol on a TS-100C thermoshaker, with centrifugation of the sample each time (10 min at 12,000× *g*, 4 °C). The supernatants were combined and the total extract was evaporated to dryness in a Labconco CentriVap vacuum evaporator (Kansas City, MO, USA). The air in the microtubes was replaced with gaseous nitrogen, and they were placed in a freezer at −80 °C for storage until analysis.

The dried material was dissolved in pyridine with the internal tricosane standard (nC23, tricosane, Sigma-Aldrich, St. Louis, MO, USA). The samples were then derivatized with the sylilating agent BSTFA:TMCS, 99:1 (Sigma-Aldrich) at 90 °C for 20 min before GC-MS analysis.

### 4.5. Gas Chromatography–Mass Spectrometry (GC-MS)

An Agilent 6850 gas chromatograph under the control of MassHunter software v. 10.1 (Agilent Technologies, Santa Clara, CA, USA) was used for GC-MS analysis. Samples were injected using an Agilent 7893 autosampler. The sample was injected in the splitless mode, with an injected sample volume of 0.4 μL. Separation was performed on an Rxi5Sil MS capillary column (30 m long, 0.25 mm in diameter, stationary phase film (95% dimethylpolyoxane, 5% diphenyl), thickness 0.25 μm; Restek Corporation, Bellefonte, PA, USA). The carrier gas was helium; constant flow was 1 mL/min; and evaporator temperature was 250 °C. The column temperature regime started with an initial temperature of 70 °C and then was linearly increased at a rate of 6 °C/min to 320 °C. The chromatogram was registered using an Agilent 5975 mass-selective detector. The mass range was 50–700 *m*/*z*. The temperature of the ion source was 230 °C and that of the quadrupole mass filter was 150 °C. The chromatographic equipment of the Resource Center of St. Petersburg State University “Development of molecular and cellular technologies” was used for this research.

### 4.6. Interpretation of GC-MS Results

The analysis of the GC-MS data was performed using the PARADISe software v. 6.0.1 (Department of Food Science Faculty of Science, University of Copenhagen, Copenhagen, Denmark [44]). To identify the mass spectra, we used the Golm Metabolome Database (GMD) library (Potsdam, Germany) [45], the library of the Laboratory of Analytical Phytochemistry of the Botanical Institute of the Russian Academy of Sciences (St. Petersburg, Russia; State Assignment no. 124020100140-7) and the AMDIS 2.71 and NIST MS Search 2.4 (National Institute of Standards and Technology (NIST), Gaithersburg, MD, USA) programs, in combination with the NIST20 libraries. The identification of metabolites was performed by the similarity of the mass spectra with the library ones and by the retention indices (RIs). The RI was determined by calibration using saturated hydrocarbons.

### 4.7. Statistical Analysis

Data in Figure 1 are presented as the mean ± SE for ≥4 experiments. Statistical data processing was performed using GraphPad Prism 8.0.1 for Windows. The graphs in the figures show the average values and their standard errors. Values with different letters are significantly different at *p* < 0.05 (Tukey’s test).

Analysis of metabolomic data was performed using R 4.3.1 “Beagle Scouts” [46]. The level of metabolites was normalized to the sample median. Outliers were detected and excluded based on the Dixon test in the outliers package v. 0.15 [47]. Data were taken logarithmically and standardized. If a compound was not found in a particular sample but was present in other replicates, this was considered a technical error, and imputation was performed using the KNN (k-nearest neighbors) method via the impute package v. 1.80.0 [48]. Principal component analysis (PCA) was performed using *pcaMethods* v. 1.98.0 [49]. OPLS-DA (Orthogonal Partial Least Squares-Discriminant Analysis) was performed using the ropls v. 1.32.0 package. Factor loadings of the predictive component and VIP (variable importance in the projection) were used to assess the statistical relationship between the variables and the factor of interest [50]. Models were evaluated by R^2^Y and Q^2^Y (*p* < 0.05). Heat maps were plotted using the ComplexHeatmap package v. 2.22.0 [51]. The fgsea algorithm (v. 1.32.0) was used for metabolite set enrichment analysis (MSEA) [52]. The sets of metabolites for biochemical pathways were downloaded from the Kyoto Encyclopedia of Genes and Genomes (KEGG) database (Kyoto, Japan) [53] via the KEGGREST package v. 1.46.0 [54], using *Oryza sativa* as a reference organism. Graphs were plotted using the Cytoscape software v. 3.10.2 [55].

Growth and viability experiments as well as metabolome analysis were performed at least in four biological replicates.

## 5. Conclusions

Taken together, the obtained results indicated a correlation linking the ability to grow and further tolerance to oxygen deprivation. In rice seedlings, given their ability to germinate under submergence, this link is clearly evident, even in the early stages of development in a juvenile organ such as the coleoptile. Its elongation was proven to indicate a higher tolerance of the fast-growing rice variety. However, growth is a very complex process and is highly regulated by various internal and external factors. The simultaneous comparison of metabolic responses in coleoptiles of two rice varieties that differed in elongation rate was effective at revealing their distinctions under oxygen limitation. These mostly concerned different levels of accumulation of tricarboxylic acids of the Krebs cycle, lactate, carbohydrates and sterols. Coleoptiles of the fast-growing and more tolerant Kuban 3 variety accumulated higher amounts of tricarboxylic acids of the Krebs cycle (citrate, aconitate, 2-ketoglutarate), sugar phosphates, disaccharides, glycosides, fatty acid derivatives and sterols. Those groups of metabolites are known to be important in maintaining growth, membrane stability and viability. The slow-growing variety Amethyst was characterized by a greater abundance of carboxylates, including glycolate, glycerate, lactate, intermediates of the dicarboxylic part of the Krebs cycle (succinate, fumarate) and 2-hydroxycarboxylates, as well as phosphoric acid. These indicated the development of an energy crisis and cytosol acidification, leading to cell damage and low tolerance. The metabolic approach we applied helped to uncover an additional unexpected result, which was the accumulation of IAA in slow-growing cv. Amethyst. This outcome requires further investigation. Thus, alterations in coleoptile elongation are in accordance with the plant tolerance to oxygen shortage, as confirmed by metabolic profiling. Therefore, it seems a metabolomic approach is needed for metabolic phenotyping (chemotyping) in the large-scale screening of newly developed varieties with a higher tolerance to oxygen deprivation.

## Data Availability

Data are contained within the article and Supplementary Materials.

## Supplementary Materials

The following supporting information can be downloaded at https://www.mdpi.com/article/10.3390/ijms252413256/s1.
