# Supplementary material for: Alterations in the Rice Coleoptile Metabolome During Elongation Under Submergence Stress
Source: Int J Mol Sci. 2024 Dec 10;25(24):13256. doi: 10.3390/ijms252413256 (PMC11678009; doi:10.3390/ijms252413256)
Supplement: Supplementary file 1 [file ijms-25-13256-s001.zip › IJMS-Yemelyanov ea-Supplementary_Figures.pdf]

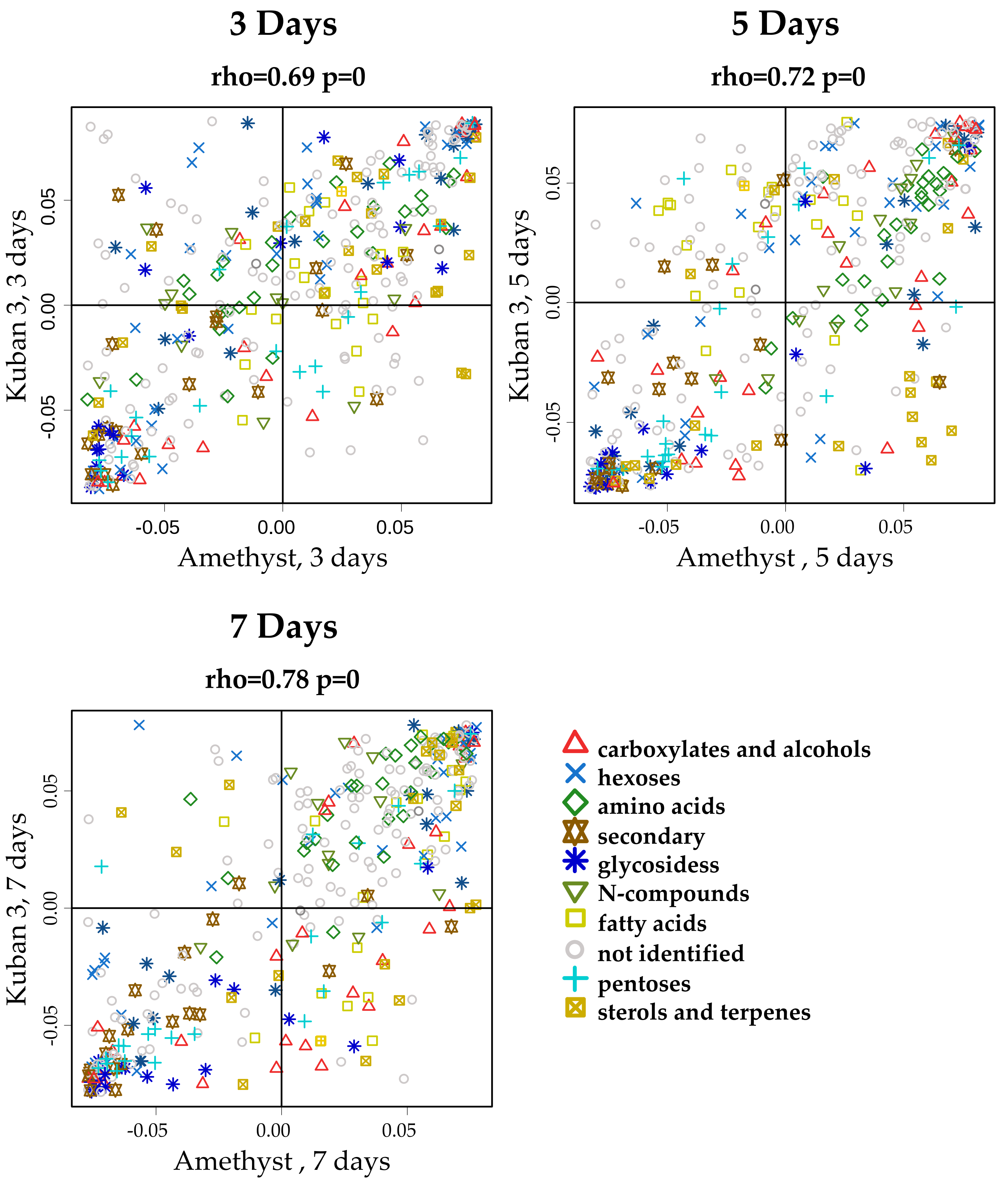

Figure S1. Comparison of the effects of hypoxia in two varieties. SUS-plot (Shared and Unique Structures) plot: scatter of metabolites in the space of loadings of predictive components derived from OPLS-DA.
rho - Spearmans' correlation.

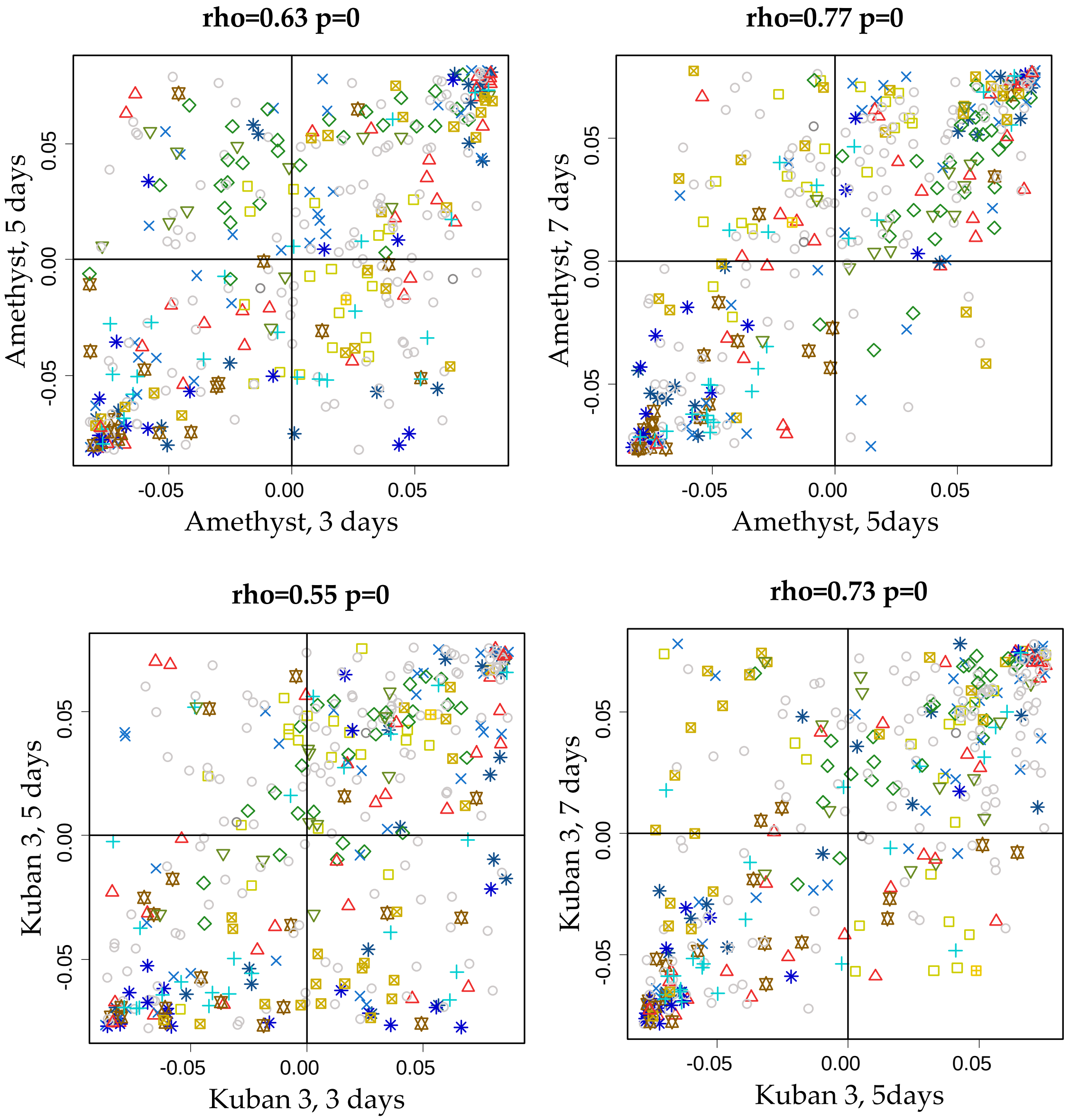

Figure S2. Comparison of the effects of hypoxia at three time points for two cultivars. SUS-plot (Shared and Unique Structures) plot: scatterplot of metabolites in the load space of predictive components derived from OPLS-DA. rho - Spearmans' correlation.

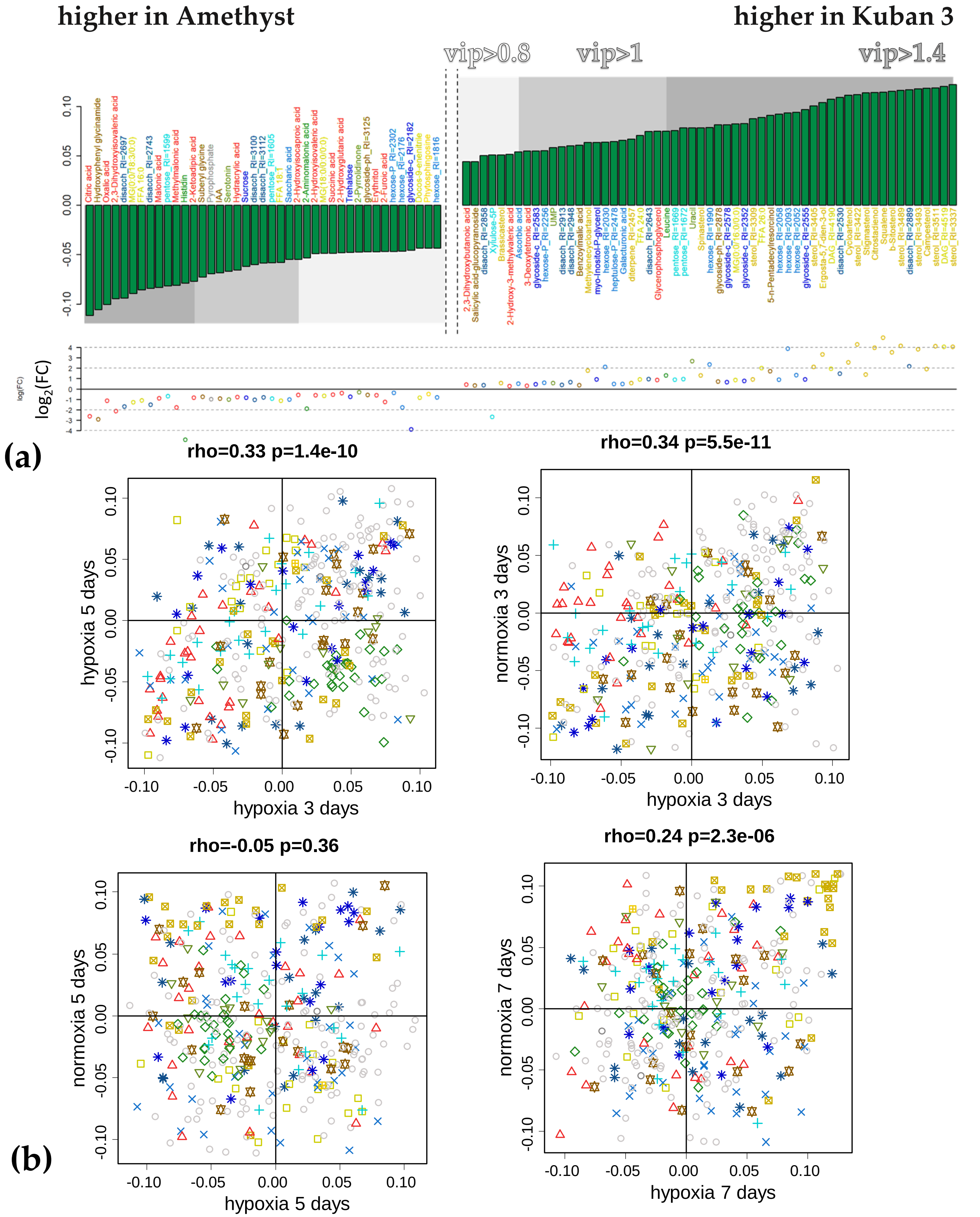

Figure S3. Analysis of the differences between two varieties.
(a) – Metabolites differently accumulated after 7 days of hypoxia in two cultivars. Barplots of factor loadings of the predictive components from OPLS-DA models. Scattered plot – log2(FC(hypoxia/normoxia)).
(b) – Comparison of differences of two varieties under hypoxia and normoxia conditions at different timepoints. SUS-plots (Shared and Unique Structures) plots: dispersion of metabolites in the load space of predictive components derived from OPLS-DA. rho – Spearmans' correlation.
